# Protective Role for the Disulfide Isomerase PDIA3 in Methamphetamine Neurotoxicity

**DOI:** 10.1371/journal.pone.0038909

**Published:** 2012-06-08

**Authors:** Gurudutt Pendyala, Carly Ninemire, Howard S. Fox

**Affiliations:** Department of Pharmacology and Experimental Neuroscience, University of Nebraska Medical Center, Omaha, Nebraska, United States of America; Okayama University Graduate School of Medicine, Dentistry and Pharmaceutical Sciences, Japan

## Abstract

Methamphetamine abuse continues to be a worldwide problem, damaging the individual user as well as society. Only minimal information exists on molecular changes in the brain that result from methamphetamine administered in patterns typical of human abusers. In order to investigate such changes, we examined the effect of methamphetamine on the transcriptional profile in brains of monkeys. Gene expression profiling of caudate and hippocampus identified protein disulfide isomerase family member A3 (PDIA3) to be significantly up-regulated in the animals treated with methamphetamine as compared to saline treated control monkeys. Methamphetamine treatment of mice also increased striatal PDIA3 expression. Treatment of primary striatal neurons with methamphetamine revealed an up-regulation of PDIA3, showing a direct effect of methamphetamine on neurons to increase PDIA3. *In vitro* studies using a neuroblastoma cell line demonstrated that PDIA3 expression protects against methamphetamine-induced cell toxicity and methamphetamine-induced intracellular reactive oxygen species production, revealing a neuroprotective role for PDIA3. The current study implicates PDIA3 to be an important cellular neuroprotective mechanism against a toxic drug, and as a potential target for therapeutic investigations.

## Introduction

Methamphetamine (METH) is a derivative of amphetamine with increased central nervous system (CNS) potency. METH abuse continues to ravage the US as well as countries around the world. While taken for its effects on the CNS, METH has significant neurotoxic properties. Several cellular and molecular mechanisms are linked to METH associated neurotoxicity such as oxidative stress, DNA damage, excitotoxicity, disruption of the blood brain barrier, and microglial activation (reviewed by [1], [2]). While many of these effects have been described in rodents, a number of studies on METH have been performed in monkeys analyzing its toxic effects on brain monoamine dopamine (DA) and serotonin (5-HT) neurons, focusing on the end-effects in the striatum [3], [4], [5], [6] with similar findings found in humans [7], [8]. However in addition to damage to the termini of projecting neurons, it is clear that METH also has toxic effects on various non-DA/5-HT neurons present in the striatum and elsewhere [9].

Since METH is a significant co-morbid contributor in human immunodeficiency virus (HIV) infection throughout the world [10], we had previously performed a study on simian immunodeficiency virus (SIV) infected rhesus monkeys, comparing a number of viral and immune parameters in those receiving METH to those receiving control vehicle injections [11]. Because these animals only differed in one factor, the administration of METH, we utilized brain samples from these animals to perform expression profiling in order to gain insights into the effects of METH on the brain. The caudate and hippocampus were chosen as they are affected by METH, both through monoaminergic connections as well as being direct targets of METH neurotoxicity. We identified the mRNA for PDIA3 (also called glucose regulated protein 58, GRP58; and endoplasmic reticulum resident protein 57, ERp57) to be up-regulated in the animals treated with METH as compared to the levels found in control monkeys treated with the vehicle (phosphate buffered saline, PBS). Studies were then performed to ascertain the role of PDIA3 in METH-induced neurotoxicity.

## Materials and Methods

### Ethics statement

Materials used in these studies were from non-human primate and mouse work performed under IACUC approval (from Scripps Research Institute, 07-0067; University of Nebraska Medical Center, 08-071-EP). Animal welfare was maintained by following NIH (Public Health Service, Office of Laboratory Animal Welfare) and USDA guidelines by trained veterinary staff and researchers under Association for Assessment and Accreditation of Laboratory Animal Care certification, insuring standards for housing, health care, nutrition, environmental enrichment and psychological well-being. These met or exceeded those set forth in the *Guide for the Care and User of Laboratory Animals* from the National Research Council of the US National Academy of Sciences. All efforts were made to ameliorate suffering of animals, including use of anesthesia with ketamine, xylazine, and phenobarbital at non-human primate necropsy, and isoflurane at mouse sacrifice.

### Rhesus macaques, SIV infection and METH treatment

As described previously [11], six rhesus monkeys (*Macaca mulatta*) were infected with an *in vivo* serial passage derivative of SIVmac251 [12], [13]. At 19 weeks of infection, animals were matched for viral load, and three were treated with an escalating dose regimen of METH injected intramuscularly (5 week ramp-up to 25 mg/kg/week), METH was maintained at this level for another 18 weeks, mimicking a usage pattern in human chronic METH abusers. The other three monkeys received PBS injections on the same schedule. All animals were sacrificed at 42 weeks post infection, before the development of AIDS.

### Monkey necropsy and RNA isolation

For necropsy, animals were lethally anesthetized, intracardially perfused with sterile PBS containing 1 U/ml heparin to clear blood-borne cells from the brain, and vital organs removed for pathological and other studies. Both caudate and hippocampus were dissected from brains and snap frozen.

### Mouse METH treatment

Eight week old C57BL/6J male mice (Jackson Laboratory, Bar Harbor, ME) were subcutaneously injected with 10 mg/kg METH or saline. Mice were sacrificed by isoflurane overdose at 30, 60, 120, and 240 minutes post injection. The striatum was dissected and snap frozen in liquid nitrogen.

### RNA isolation

RNA was isolated using Trizol (Life Technologies, Carlsbad, CA) and quantified. spectrophotometrically.

### Microarray

Microarray analysis was performed using rhesus gene chips (Affymetrix, Santa Clara, CA). Briefly, 200 ng total RNA from caudate was reverse-transcribed to generate cDNA followed by *in vitro* transcription to generate biotinylated cRNA using the Affymetrix 3′IVT Express kit per manufacturer's recommendations. Biotin labeled target was hybridized for 16 hrs to rhesus arrays following standardized protocols per Affymetrix recommendations. Following hybridization, arrays were washed, stained, and scanned using the Affymetrix GeneChip System. Arrays were evaluated for quality control (QC) by a variety of metrics including background signal, 3′5′ ratios of actin and hybridization kinetics of spike-in probes per Affymetrix recommended parameters. All arrays passed QC and were subjected to further analysis. Data were analyzed using the Partek Genomics Suite (Partek, St. Louis, MO), and probe sets mapped to genes using the Database for Annotation, Visualization and Integrated Discovery (DAVID v6.7) [14]. Annotations were updated from the NCBI Gene database as of 10/16/2011. MIAME compliant microarray data have been deposited in the NCBI GEO database under accession GSE33707.

### Quantitative real-time PCR

Isolated RNA was used for reverse transcription. In brief, reverse transcription was carried out using the Superscript kit (Life Technologies) at 2 µg RNA per 50 µl reaction, for 1 h at 42°C, followed by 70°C for 5 min to inactivate the enzyme. RNase H (New England Biolabs, Beverly, MA) treatment was then performed at 37°C for 20 min. Then an equal volume of RNAse and DNAse free water was added to each reaction.

Quantitative real-time PCR (qRTPCR) was performed using gene-specific primers and probes. Primers and probe sequences were designed for rhesus using the Genescript online tool (https://www.genescript.com/ssi-bin/app/primer) obtained from Eurogentec (San Diego, CA). Primer and probe sequences for PDIA3 are: Forward-TTGCACTGCCAACACTAACA, Reverse-CTTCTTCAGGTGGCTGACAA, Probe-ACGGCGTCAGTGGATATCCAACC; for the housekeeping genes (18S, GAPDH and TBP) sequences are given in [15]. For the human and mouse PDIA3 and mouse HSPA5 genes, gene and species-specific TaqMan primers and probes (Life Technologies) were used. The above housekeeping genes for 18S and GAPDH were used since they recognized human and mouse sequences as well. For qRTPCR, in brief, 2 µL (1∶100 diluted) cDNA was used for assaying endogenous 18S ribosomal RNA; 5 µL (undiluted) each for all other genes. All reactions were performed in duplicate. 12.5 µL of platinum qPCR UDG Supermix (Life Technologies) was added per 25 µl reaction. The reaction mixture was brought to a final concentration of 5 mM MgCl_2_. Real time PCR was performed in 96-well plate on a Step One Plus machine (Life Technologies). The delta Ct method was performed to determine relative concentrations using housekeeping genes as the normalizing value. Unpaired student's t-tests, or one-way ANOVA followed by Tukey's test, were used for statistical analysis.

### Cell culture

Rat striatal neurons from embryonic day 18 Sprague Dawley rat (Brain Bits LLC, Springfield, IL) were plated at a density of 4×10^5^ cells per well onto a 6-well plate containing two coverslips per well in Neurobasal media containing B27 (Life Technologies). After 8 days *in vitro* (DIV), cells were treated with 250 µM METH for 24 h while control cells received no METH. The METH concentration and time course was determined to be the appropriate concentration on primary neurons without causing excessive death and thus allowing measurement of protein changes. After 24 h, cells were fixed in 4% paraformaldehyde and double labeling performed as described below.

Human neuroblastoma cell line SK-N-BE(2), was cultured in Dulbecco's modified Eagle's medium containing F12 supplement, 10% goat serum and GlutaMAX™-1. PDIA3 cDNA (Origene Technologies, Rockville, MD) was cloned into pcDNA 3.2/V5 (Invitrogen). For overexpression, plasmid DNA transfection was performed using Nucleofection (Lonza, Walkersville, MD) and Geneticin-resistant cells selected. For generating knockdown clones, PDIA3-specific GIPZ lentiviral shRNAmir was purchased from ThermoScientific (Huntsville, AL). Following lentiviral transduction puromycin-resistant cells were selected.

### Cell toxicity assay

SK-N-BE(2) cells plated at a density of 5×10^4^ per well in a 24-well plate were treated with 500 µM METH for 48 hours. Again the METH concentration and time course was determined to be appropriate for this cell line without causing excessive death, yet able to produce a measurable effect. Cell toxicity was determined by measuring lactate dehydrogenase (LDH) release from treated cells using the LDH assay kit (Roche, Indianapolis, IN) as per the manufacturer's instructions. In brief, the culture medium from cells was added to the enzymatic reaction buffer and incubated for 30 min at room temperature. Absorption values at 490 nm were measured using a plate reader to determine levels of released LDH. Culture medium from cells treated with 2% Triton-X 100 was used as a positive control.

### Immunofluorescence

Double labeling was performed on both the control and METH treated rat striatal neurons. Cells were permeabilized with 0.25% Tween-20 in PBS for 20 min at room temperature and washed twice with PBS. Cells were incubated in 10% normal goat serum with 0.25% Tween-20 in PBS for 30 min at room temperature and then incubated with anti-PDIA3 (1∶100, Assay Designs, Plymouth Meeting, PA) at 4°C overnight. After incubation with fluorescence-labeled secondary antibody (1∶100, goat anti-rabbit IgG, Invitrogen) for 1 h at room temperature, the second primary antibody anti-MAP2 (1∶500, Sternberger Monoclonals, Lutherville, MD) was added at 4°C overnight. After incubation with second secondary antibody (1∶100, chicken anti-mouse IgG, Life Technologies), cells were washed and mounted with Prolong Gold with DAPI and analyzed by microscopy (Carl Zeiss, Thornwood, NY).

For immunoflourescent labeling on parental, siRNA and overexpression clones of SK-N-BE(2) cells, cells were permeabilized with 0.25% Tween-20 in PBS for 20 min at room temperature and washed twice with PBS. Cells were incubated in 10% normal goat serum with 0.25% Tween-20 in PBS for 30 min at room temperature and then incubated with anti-PDIA3 (1∶100, Assay Designs) at 4°C overnight. After incubation with secondary antibody (1∶100, goat anti-rabbit IgG, Invitrogen) for 1 h at room temperature, cells were mounted with Prolong Gold with DAPI and analyzed by microscopy (Carl Zeiss).

### Western Blot Analysis

Cells were harvested using ice cold PBS, pelleted, and lysed in radioimmunoprecipitation assay buffer (ThermoScientific) with 1× protease inhibitors (Roche). Samples were sonicated and measured for protein concentrations with the bicinchoninic acid assay (ThermoScientific). 10 µg of protein was loaded into a 4–12% BisTris gel (Invitrogen), electrophoresed, and transferred to a nitrocellulose membrane (Invitrogen) using iBlot (Invitrogen). Membrane was incubated in SuperBlock (ThermoScientific) for 1 hour at room temperature and then overnight in anti-PDIA3 (1∶1000 Assay Designs) or anti-Actin (1∶7500 Sigma-Aldrich, St. Louis, MO) at 4°C. Membrane was washed three times with 1× Tris-buffered saline and 0.1% Tween-20 followed by incubation with secondary antibody (1∶10000 Anti-rabbit HRP, ThermoScientific) for 1 hour at room temperature. Membrane was washed as above and incubated for five minutes in 1∶1 West Pico chemiluminescent substrate (ThermoScientific) and the signal was detected and quantified using Gel Logic Imaging System (Carestream, Woodbridge, CT).

### Reactive oxygen species (ROS) assay

Intracellular production of ROS was assessed by 2′,7′-dichlorfluorescein diacetate (DCFH-DA) oxidation. Clones over expressing and knock down for PDIA3 along with the parental cells were treated with 500 µM METH for 1 h and then incubated with 20 µM DCFH-DA (Sigma) for 30 min. After incubation cells were washed with PBS and the fluorescence visualized at 495 nm for excitation and 529 nm for emission using a fluorescence plate reader.

### Statistics

All data represented were analyzed from three independent experiments. Group comparisons were performed using the tests described in the text and figure legends. Differences were considered significant at p<0.05. Tests were performed using Excel (Microsoft Corporation, Redmond, WA) and Prism software (GraphPad Software Inc., San Diego, CA) for Macintosh.

## Results

We initiated our studies by performing expression profiling on the caudate and hippocampus (targets of the nigrostriatal and mesolimbic dopaminergic systems, respectively) of the SIV infected monkeys, treated or not with METH, using a rhesus-specific microarray platform. Based on a p value of <0.01 and a fold change of greater than two, 50 genes were changed in expression in the caudate and 49 in the hippocampus. Of these, ten protein-coding genes were found to be significantly changed in both, all of which were up-regulated (Table 1).

**Table 1:** List of genes up-regulated in both the caudate and hippocampus from SIV infected, METH and PBS administered monkeys.

Gene Symbol	Gene Name	Caudate		Hippocampus	
		Fold	p value	Fold	p value
FGF12	fibroblast growth factor 12	2.11	0.0058	2.11	0.0045
GLG1	golgi glycoprotein 1	2.41	0.0014	2.18	0.0027
LOC702990	microtubule-associated tumor suppressor 1 homolog	2.89	0.0054	2.67	0.0004
LOC715864	CUGBP Elav-like family member 4-like	2.46	0.0067	2.46	0.0041
OAZ2	ornithine decarboxylase antizyme 2	2.58	0.0002	2.12	0.0019
PDIA3	protein disulfide isomerase family A, member 3	4.92	0.0057	2.42	0.0056
PRKX	protein kinase, X-linked	2.99	0.0074	2.09	0.0000
TBL1X	transducin (beta)-like 1X-linked	2.09	0.0030	2.68	0.0004
TMBIM1	transmembrane BAX inhibitor motif containing 1	3.67	0.0029	2.25	0.0001
TST	thiosulfate sulfurtransferase	2.63	0.0012	2.39	0.0053

The gene symbol, gene name, p-value (unpaired Student's t-test) and fold change between the groups is indicated. Genes with p<0.01 and a fold change of >2 in both regions are shown.

We focused on protein disulfide isomerase family member A3 (PDIA3) given its neuroprotective implications. One study implicated a protective role for PDIA3 against neurotoxicity induced by prion infection [16]. A similar neuroprotective role for PDIA3 has been documented against β-amyloid aggregation in Alzheimer's disease [17]. Moreover, a common chord that connects these neurodegenerative disorders is cellular stress including generation of reactive oxygen species (ROS) and endoplasmic reticulum (ER) stress. PDIA3 is localized in the ER lumen and an earlier study demonstrated its induction during ER stress [18]. Since METH has been shown to induce ER stress and subsequent neuronal death [19], we hypothesized that PDIA3 may be up-regulated to counter METH induced neurotoxicity.

**Figure 1:**
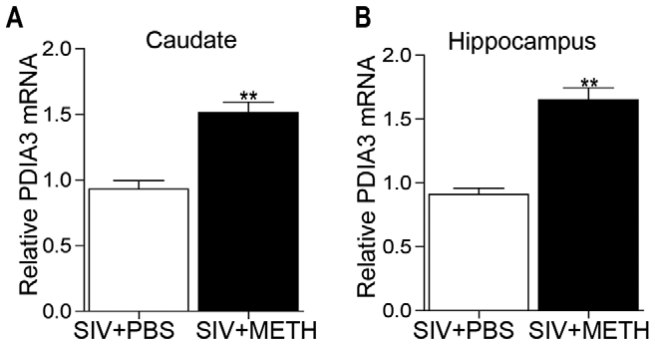
PDIA3 is increased in by METH in monkeys *in vivo*. Levels of PDIA3 mRNA expression from qRTPCR in brain regions from the two groups of animals, differing only by METH treatment. (A) caudate and (B) hippocampus. **p<0.01, unpaired Student's t-test.

**Figure 2:**
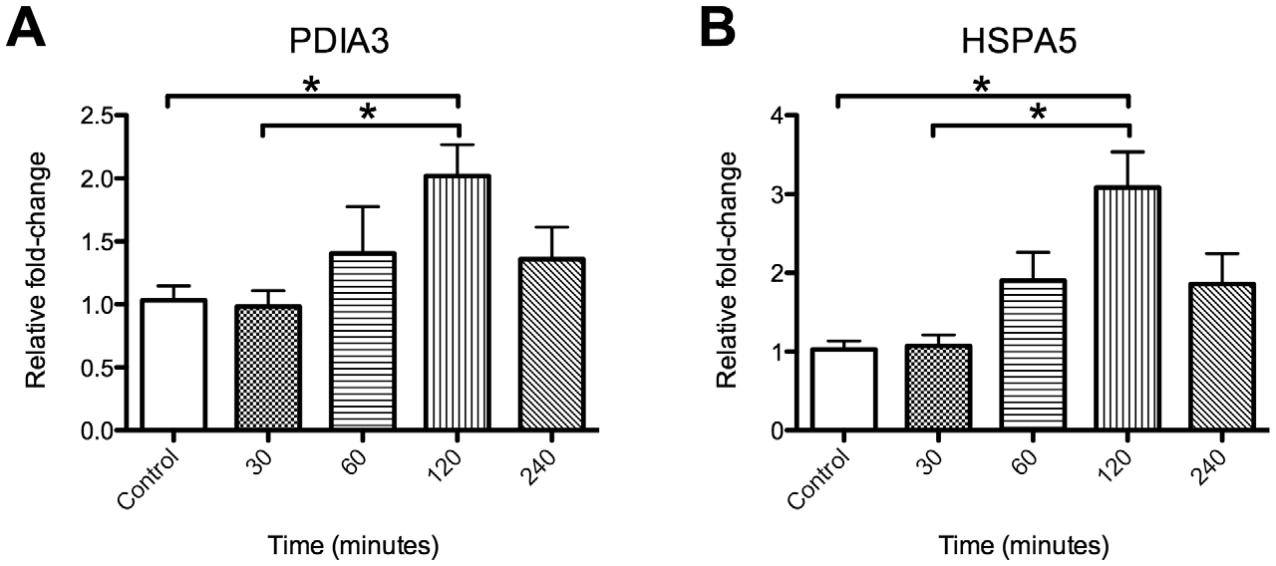
PDIA3 is increased by METH in rodents *in vivo*. Time course of gene expression, determined by qRTPCR, in striatum from mice treated with 10 mg/kg METH at the indicated time points. (A) PDIA3 (B) HSPA5 (as a positive control). *p<0.05 as determined by a one-way ANOVA followed by a post hoc Tukey's test.

We first validated the increase in PDIA3 in monkey striatum by quantitative real time polymerase chain reaction, revealing a significant increase in PDIA3 mRNA expression in the METH treated monkeys compared to the animals not treated with METH (Fig. 1). Since all monkeys were SIV infected, we next wanted to assess whether METH alone is capable of inducing PDIA3. Single dose injections of METH in mice are informative in determining the direct effect of METH on gene expression [19], [20], [21]. We therefore administered METH to mice and did a time course experiment for striatal gene expression, examining PDIA3 and, as a positive control, HSP5A (also known as GRP78/BiP), found by others to be induced by METH in mice [19]. Indeed both genes were induced by METH (Fig. 2), revealing that METH itself induces PDIA3 *in vivo*.

**Figure 3:**
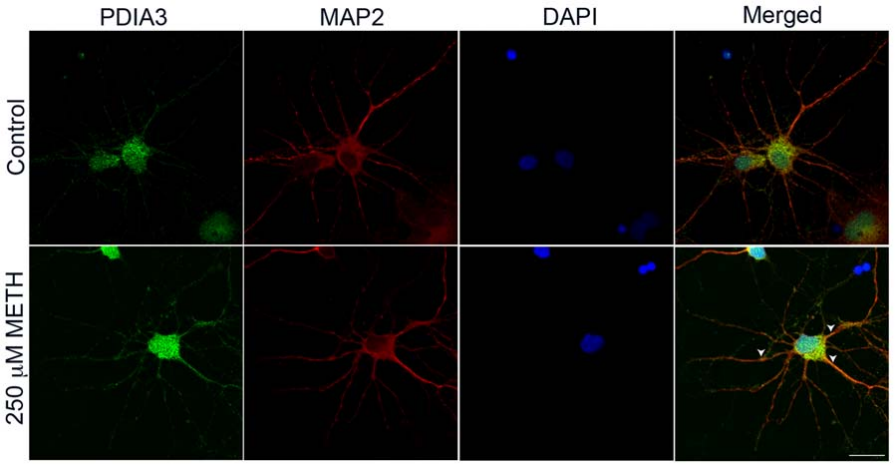
METH induction of PDIA3 *in vitro* in rodent neurons. Primary rat striatal neurons (8 DIV) stained for levels and distribution of PDIA3 after 250 µM METH treatment for 24 h along with MAP2 staining for neuronal structure and DAPI for cell nucleus. Control cells show basal levels of PDIA3 while cells treated with METH show an increase and redistribution of PDIA3 along the neuronal processes indicated by arrowheads. Scale bar = 10 µm.

We next performed studies on METH-treated neurons *in vitro* in order to examine whether METH itself can increase the PDIA3 at the protein level. Treatment of striatal neurons with METH resulted in an increase in PDIA3 expression in neurons, evident in both the soma and neuronal processes (Fig. 3). Thus not only does METH treatment *in vivo* lead to increased PDIA3 expression, METH treatment *ex vivo* reveals a direct distinct response in the neurons through up-regulation of PDIA3.

**Figure 4:**
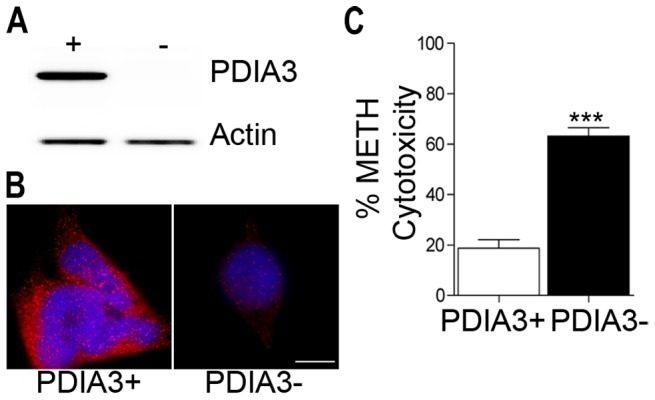
Increased PDIA3 protects from METH toxicity. Examination of SK-N-BE(2) cells for expression and knockdown for PDIA3 by (A) western blot and (B) immunofluorescence. Scale bar = 10 µm. (C) Increased METH cytotoxicity in PDIA3 knockdown cells compared to the PDIA3 expressors. Cytotoxicity was assessed by a lactate dehyrogenase assay following 48 hrs exposure to 500 µM METH. ***p<0.001, unpaired Student's t-test.

To examine whether the increase in PDIA3 is neuroprotective, we utilized SK-N-BE(2) neuroblastoma cells, chosen for their sensitivity to METH-mediated neurotoxicity. PDIA3 was constitutively expressed as well as knocked down in these cells (Fig. 4A and B). Cytotoxicity assays revealed that knockdown of PDIA3 resulted in significantly increased METH-induced cell death (p<0.001) compared to the PDIA3 expressing cells (Fig. 3C).

**Figure 5:**
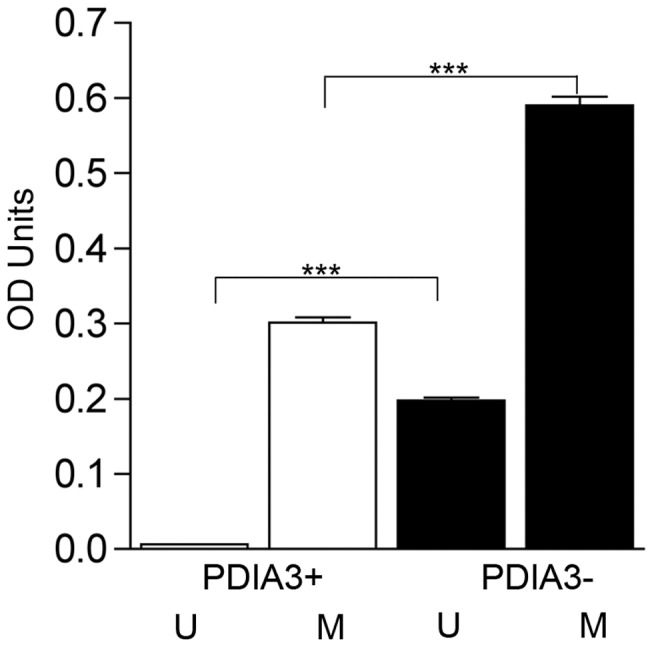
Increased PDIA3 suppresses ROS production. Cells treated with 500 µM METH for 1 h were assessed for production of ROS using DCFH-DA assay. A significant increase in ROS production is seen in cells knocked down for PDIA3, compared to PDIA3 expressors, both with and without METH treatment. Two-way ANOVA p<0.0001, Bonferroni post-tests. ***p<0.001. U – No treatment; M – METH treatment.

Generation of intracellular ROS is a critical contributor in METH mediated cell death [1]. To further ascertain a potential neuroprotective role for PDIA3, we performed an intracellular ROS assay on these cells. A significant increase in intracellular ROS was observed in cells knocked down for PDIA3 both in the presence and absence of METH (p<0.001) compared to the PDIA3 expressing cells (Fig. 5). Together, these data demonstrate a neuroprotective role for PDIA3 during METH cytotoxicity.

## Discussion

Using two groups of monkeys differing only by METH administration, we found PDIA3, an endoplasmic reticulum (ER) resident thiol disulfide oxidoreductase and a molecular chaperone, to be significantly up regulated after METH treatment. Indeed treatment of mice and of primary striatal neurons with METH resulted in increased PDIA3 expression, revealing its direct response to METH.

PDIA3 has been linked to numerous human disease states. Expression of PDIA3 is increased in transformed cells, and it is thought that the role it plays in oncogenic transformation is directly due to its ability to control intracellular and extracellular redox activities through its thiol-dependent reductase activity [22]. An increase in PDIA3 expression has also been observed in the early stages of prion disease, suggesting that it may play a neuroprotective role in the cellular response to prion infection [16]. It has been documented that METH mediates ER stress in the striatum [19] thus an increase in stress response proteins would be expected. We hypothesized a similar neuroprotective effect of PDIA3 in our current study and our cumulative data revealed a similar neuroprotective effect of PDIA3 against METH toxicity.

The initial gene array expression profiling studies were carried out in groups of SIV infected monkeys, differing only by the administration of METH during the stable phase of infection. This is in one sense a limitation of our study, in that the initial discovery portion of the study was performed from tissue from this study. While having uninfected animals, treated or not with METH, would have been ideal, the use of these animals allowed an initial discovery based approach to factors induced by METH in the brain. Therefore we utilized mice to assess PDIA3 response to a METH only *in vivo* scenario, confirming the METH-induced PDIA3 found in the SIV infected monkeys. An additional advantage of the initial monkey studies were its well-controlled nature and dosing typical of human abuse was a distinct advantage, and our subsequent studies on primary neurons and the neuroblastoma cell line also revealed the effect on PDIA3 to be due to METH itself.

It is possible that the up-regulation of PDIA3 could be an early protective event preceding eventual METH mediated neurotoxicity, similar to the increase of PDIA3 expression found early in the course of prion disease before the extensive cell death observed in the later stages of prion toxicity [16]. However while molecular and imaging studies reveal a number of abnormalities in chronic METH users, some of these are reversible with abstinence, and in general the neuronal damage spares the cell bodies [23]. Thus increased PDIA3 expression may be one of the mechanisms induced to protect the neuron during METH use, enabling the cell to attempt reparative processes during abstinence. Further *in vivo* studies in model systems would allow the examination of this possibility.

In summary, these studies reveal PDIA3 to be an important cellular neuroprotective mechanism against a toxic drug, and implicate PDIA3 having a role in a more general neuroprotective pathway and as a potential target for therapeutic investigations.
